# Integrating phenotypic, molecular, and bioinformatics approaches for developing drought-tolerant sesame

**DOI:** 10.1038/s41598-025-06891-0

**Published:** 2025-07-01

**Authors:** Lamyaa M. Sayed, Khaled Adly Mohamed Khaled, Ghada M. Samaha, Ayman Anter Saber

**Affiliations:** 1 Genetics Department, Faculty of Agriculture, Ain Shams University, Cairo, Egypt; 2 Genetics Department, Faculty of Agriculture, Beni-Suef University, PO box, Beni Suef, 62517 Egypt; 3 Field Crops Research Department, Agricultural and Biological Research Institute, National Research Centre, Dokki, Giza, Egypt

**Keywords:** Abiotic stress, Crop improvement, Sesame, Drought tolerance, Genetic stability, SCoT molecular markers, Bioinformatics, Biotechnology, Genetics, Plant sciences

## Abstract

Breeding climate-resilient crops with high tolerance to abiotic and biotic stresses represents a major challenge in responding to climate change. The present study evaluated new sesame lines under drought conditions, which required the implementation of multi-environment experiments (MET) with the aim of identifying the most productive and stable sesame lines combining phenotypic evaluation, SCoT-PCR markers, and bioinformatics analysis. Seed yield was assessed across 18 limited irrigation environments, revealing significant genotype-by-environment interactions. Four stable, high-yielding lines (C5-8, C6-9, C6-11, and C9-3) were identified under optimal and drought conditions using parametric and non-parametric statistics, AMMI, and GGE biplot analysis. SCoT-PCR analysis revealed a reasonable level of genetic diversity among the genotypes, with primer SCoT-21 showing the highest polymorphism (88.24%). Bioinformatics analysis of SCoT-21 and 28 amplified regions identified potential genes associated with drought tolerance, including a DNA repair helicase XPD gene and a malonyl-coenzyme: anthocyanin 5-O-glucoside-6′′′-O-malonyl transferase-like gene. These findings provide valuable insights for developing drought-resistant sesame varieties and highlight the power of integrating phenotypic, molecular, and Bioinformatics approaches in crop improvement.

## Introduction

Sesame is among the earliest cultivated oil crops, prized for its nutrient-rich seeds, which contain 44–57% oil, 13–14% carbohydrates, and 18–25% protein^1,2^. The high quality of sesame oil is attributed to its rich concentration of antioxidants like sesamin, sesamolin, and sesamol, along with unsaturated fatty acids such as linoleic and oleic acids^3,4^. Beyond oil production, sesame seeds are widely used in various culinary applications, from garnishes to pastes^4^.

Sesame is a significant crop for Egypt due to its suitability for sandy soils, low water and fertilizer requirements, and its status as a valuable industrial crop^5,6^. However, sesame cultivation in Egypt faces challenges, including a lack of commercial varieties, inconsistent yields, fungal diseases, competition from other crops (like maize and rice), and Environmental stressors like high salt concentration and water deficit^5,6^. This is particularly concerning given that Egypt relies heavily on imports to meet its vegetable oil needs. Sesame is relatively tolerant to dehydration and drought due to its strong root system. However, drought stress restricts sesame growth and development and adversely affects the quality and quantity of its yield^7^. Some study focused on developing new drought-resistant sesame varieties to combat yield losses caused by drought, a problem exacerbated by climate change^8–10^. The water deficit negatively impacts all growth stages—from physiological and biochemical to the morphological and molecular—leading to significant yield reductions or complete crop failure^11,12^. However, the variation in physiological responses to drought among different sesame genotypes^13^ provides an opportunity for developing drought-resistant varieties.

Breeding for stress resistance is crucial for sustainable agriculture in the face of climate change^14,15^. To achieve this, it is essential to evaluate the performance of new varieties across diverse environments to assess their stability and understand how genotype-by-environment interactions (GEI) influence traits like seed productivity^16,17^. GEI significantly impacts the average performance of genotypes, making stability analysis through multi-environment trials (METs) a vital tool for evaluating genotype performance. Integrating multiple experiments through multi-environment trial (MET) analysis provides valuable breeding parameters and enhances the understanding of genotype-by-environment interactions, making it a critical tool for crop improvement programs^18,19^ .

Statistical techniques like the AMMI model^20^ and GGE biplot analysis^21^ are valuable for studying GEI. Furthermore, genetic stability can be estimated using both parametric methods, which rely on assumptions about data distribution^22^, and non-parametric methods, which are based on data rank^23^. This study employs a combination of these methods for a more accurate estimation of genetic stability.

Many approaches were integrated to study abiotic stress like Panomics. Panomics refer to comprehensive integrated analysis of multiple ‘omics’ layers (e.g., genomics, transcriptomics, proteomics, metabolomics, phenomics, ionomics, etc.) to understand complex biological mechanisms (e.g., those associated with stress tolerance)^24^. Reference^8^ mentioned that integrating panomics with advanced tools delivers novel insights for breeding stress- smart crops. They demonstrated that future directions should prioritize field-level validation of panomics data, leverage single-cell and spatial omics, and implement precise cell/tissue-specific phenotyping along with AI-driven models. Further, they highly recommend engineering ‘full gene package’ approach and leveraging synthetic biology for designing ‘genetic circuits. Moreover, exploring crop wild relatives and epigenome editing represent new research opportunities for advancing combined stress tolerance. Al- though traditional panomics have advanced single-stress research, their application to combined abiotic stresses is still inadequate.

By integrating traditional and modern breeding approaches, could be efficiently develop superior sesame lines with improved traits^25,26^. This is further supported using molecular markers, which provide valuable insights into genetic diversity and are crucial for optimizing plant utilization in response to climate change^27,28^. Molecular methods were used to pinpoint genes involved in a plant’s response to environmental stressors like drought or extreme temperatures^29^. To find these genes and their locations on the plant’s DNA, researchers employ techniques like SCoT, SSR, and AFLP markers^30–33^. DNA-SCoT has proven effective in identifying changes in gene activity, stress tolerance levels, and the overall genetic stability of plants^30,32,34,35^.

Genetic diversity has a vital role in sesame adaptability to drought conditions. The use of SCoT markers to estimate genetic similarities and clustering patterns confirms the importance of diverse germplasm resources.

SCoT markers have high polymorphism in wheat (55.56%), effectively detecting precise genetic differences among close cultivars^36^. Their functional link genetic diversity to agricultural important traits like drought tolerance^37,38^. They distinguish cultivars into geographic or evolutionary clusters, as seen in wheat from Egypt, Sudan, and Mexico, so in breeding programs, SCoT markers aid in selecting diverse parents for heterosis for accelerating variety development^39^.

Crop improvement has been turned on by the combination of bioinformatics and molecular breeding, which offers effective methods and instruments for creating more flexible, nutrient-rich, and productive crop types^40^. These methods have made it possible to identify new genetic markers and candidate genes for important agronomic traits depending on genomic sequencing developments, data management, and analytical tools^41^. They have also made it possible to design and execute efficient and successful breeding strategies, including genome editing, genomic selection, and marker-assisted selection, resulting in notable progress in crop production, quality, and adaptation to biotic and abiotic challenges^42^.

The objectives of this study were to identify the best sesame genetic resources under drought conditions to be nominated for initial crop trials in multiple sites and years as a basis for releasing new varieties and use SCoT-PCR markers bioinformatic analysis for phylogenetic relationships to understand the relationships between 30 sesame genotypes under drought stress to develop a hybridization program that contained genetic distant parents to increase resilience to climatic change.

## Material and methods

### Experimental sites

Beni Suef, North Central Egypt; Giza, North Egypt; and Nubaria, West Delta, Egypt, was the site of the trials during the four summer seasons (2020 to 2023). Planting took place in May and harvesting in September. Each study site’s characteristics are displayed in Table 1. Surface irrigation was applied in Beni Suef and Giza, whereas drip irrigation was used in Nubaria.

**Table 1:** Characterization of the study sites.

		Beni Suef (Northern Central Egypt)	Giza (Northern Egypt)	Nubaria (western delta of Egypt)
Geographical description	Latitude (N)	29° 4′ 0″ N,	29.9870°N	30° 36′ 36″ N,
	Longitude (E)	31° 5′ 0″ E	31.2118°E	30° 25′ 48″ E
Soil properties	Ph	8.0	8.3	8.7
	Texture	Clay	clay	Sandy
	Organic matter content (OMC) %	1.45	1.15	0.40
	EC (dS m^-1^)	1.4	0.50	0.44
	Available water (%)	26.0	22.0	7.0
	Field capacity (%)	42.0	40.1	4.2%
	Wilting point (%)	30.0	20.0	0.70

### Plant material

Twenty-eight new sesame lines (C1-5, C1-6, C1-8, C1-9, C2-2, C2-3, C2-6, C3-4, C3-7, C3-8, C5-7, C5-8, C6-2-C6-7, C6-9, C6-11-C6-12, C8-4, C8-8, C8-11, C9-3, C9-6-C9-7, C9-15, C9-20) were obtained from the Department of Agronomy, Faculty of Agriculture, Cairo University, Egypt^43^. Two commercial cultivars were used as control, namely Shandweel (C1) and Sohag (C2), obtained from the Egyptian Ministry of Agriculture and Land Reclamation.

### Experimental design and agricultural practices

Genotypes were arranged in a randomized complete block design with four replicates in a plot consisting of three 3-m rows with a spacing of 60 cm between rows and 10 cm between plants. Weed control was carried out manually twice, the first after thinning and the second three weeks later. Thinning was done when the plant has 4–6 leaves, leaving one plant in the hole.

Phosphate fertilization: The acre needs (200) kg of single super phosphate added when preparing the land for cultivation. Nitrogen Fertilization: 10 kg of nitrogen per acre was added immediately after planting, followed by 20 kg of nitrogen per acre immediately after thinning. Potassium fertilization: 50 kg of potassium sulfate (48% K2O) was added at once after thinning. Micronutrients: The plants were sprayed with a mixture of chelated micronutrients (60 g zinc + 40 g iron + 50 g manganese + 20 g copper) when the plant reached 40 cm in height, and the second dose was given three weeks later.

Irrigation: At the Beni Suef and Giza sites (clayey soil) where surface irrigation was applied, under drought conditions (50% irrigation) irrigation was done three times during the first 60 days of planting, stopped at the flowering stage (at 60 days of age), while under optimum conditions the number of irrigations was five (100% irrigation). At the Nubaria site, a sandy soil, irrigation and fertilization were applied weekly, eight doses of fertilization were added and irrigation was stopped at the flowering stage (at 60 days of age), which represented drought conditions (50% irrigation), while under optimum conditions (100% irrigation) it continued until the appearance of ripening signs. Table 2 showed applied irrigation water (m3 ha⁻^1^ season⁻^1^) under 100% and 50%irrigation (IRg).

**Table 2:** Applied irrigation water (m3 ha⁻^1^ season⁻^1^) under 100 and 50% irrigation (IRg).

	100% irrigation				50% irrigation			
Season	2020	2021	2022	2023	2020	2021	2022	2023
Beni Suef site (Surface irrigation)	5225	5225	5300	5350	2664.7	2664.7	2703	2728.5
Giza site (Surface irrigation)	3918	4000	3975	4012	1998	2000	2027	2046
Nubaria site (Drip irrigation)	3770	3780	3760	3800	1922.7	1927.8	1917.6	1938

(IRg): Represents the irrigation rate (mm).

Harvest: When the leaves turn yellow and fall, and the lower capsule on the stem turns yellow.

The volume of irrigation water calculation used the formula:

$${\text{IRg }} = \, \left( {{\text{ETO }} \times {\text{ Kc}}} \right) \, /{\text{ Ei }} - {\text{ R }} + {\text{ LR}}$$

where IRg represents the irrigation rate (mm), ETO is reference evapotranspiration (mm/day) (estimated from the Central Laboratory for Climate -Agricultural Research Centre, Egyptian Ministry of Agriculture at three sites, Kc is the crop factor, Ei is the irrigation efficiency, R is the rainfall (mm) and Penman–Monteith equation helped compute the irrigation water requirements, and LR is the amount of water required for salt leaching (mm).

### Statistical analysis

Gene State software was utilized to do an analysis of variance (ANOVA), and determine the GEI (genotype—environmental interaction) through a combined analysis as well as simple correlation coefficients. We investigated genotypes by environment interaction (GEI) and genotype stability using additive main effects and multiplicative interaction (AMMI) calculated according to^44^ to determine G, E and G × E effects on the seed of yield. The equation that was employed was this one:

$$Y_{ger } = \mu + \gamma_{g} + \beta_{e} + \mathop \sum \limits_{n - 1}^{N} P_{n} Y_{gn} S_{en} + \theta_{ge} + \varepsilon_{ger}$$

where the observed value (Y_ger_) for a given genotype (g) in environment (e) and replicate (r) is modelled as a function of the grand mean (μ), the deviation of the genotype means from the grand mean (ɣ_g_), the deviation of the environment means from the grand mean (β_e_), and a residual (Ɵ_ge_) which includes the genotype-by-environment interaction. Ɛ_ger_ represents the difference between the observed value and the mean for that specific genotype-environment combination. Principal component analysis (PCA) is employed to decompose the genotypic and environmental effects, using eigenvalues (Ƿ_n_) to represent the variance explained by each principal component (n), and PCA scores (Ƴ_gn_ and Ƨ_en_) to quantify the contribution of each genotype and environment to those principal components.The genotype’s main effect and genotype by environment interaction (GGE biplot) calculated according to^45^ to detect the genotype by environment interaction pattern, using stability and mean yield for genotypes selection. The model that was employed was:

$${\mathbf{Y}}_{{\varvec{i}}{\varvec{j}}}-\boldsymbol{ }{\overline{\mathbf{Y}} }_{{\varvec{j}}}=\boldsymbol{ }{{\varvec{\lambda}}}_{1}{{\varvec{\xi}}}_{{\varvec{i}}1}{{\varvec{\eta}}}_{{\varvec{j}}1}+\boldsymbol{ }{{\varvec{\lambda}}}_{2}{{\varvec{\xi}}}_{{\varvec{i}}2}{{\varvec{\eta}}}_{{\varvec{j}}2}+\boldsymbol{ }{{\varvec{\varepsilon}}}_{{\varvec{i}}{\varvec{j}}}$$

where: i refers to genotype, j to environment. Y_ij_ to average of i in j; Ȳ_j_ to average of the characters over genotypes in j’s mean effect; λ_1_, λ_2_ to values related to PC1 and PC2 as single value; ξ_i1_, ξ_i2_ to eigen of i for PC1 and PC2 respectively, ɳ_j1_ and ɳ_j2_ = eigen of j for PC1 and PC2 respectively, Ɛ_ij_ = residual linked to i in j.

### Genomic DNA isolation

Five seedlings were planted at the Laboratory in the soil of nursery and leaf samples from every 2–3 weeks old seedling were collected for genomic DNA isolation. Fresh leaf samples were immediately dried in liquid nitrogen and ground into a fine powder with a mortar and pestle. The DNA extraction process was carried out using the “i-genomic DNA” plant DNA Extraction Mini kit (iNtRON Biotechnology, Inc., Korea) according to the manufacturer instructions. Genomic DNA was loaded in 0.8% agarose gel and separated by electrophoresis for 60 min at 100 V.

### SCoT-PCR reactions

Ten SCoT primers (Table 3) were synthesized by (Willowfort, UK). The reaction conditions were optimized and mixtures (10 μl total volume) contained 1 μl of DNA template, 1 μl of primer, 5 μl of Master-Mix (Cosmo, Sigma) and sterile ddH_2_O. Amplification was performed on a Lab cycler thermal cycler (Sensoquest, Germany), programmed for 37 cycles as follows: Initial denaturation, 94 °C/4 min (one cycle), denaturation, 94 °C/1 min, annealing 50 °C /45 s, extension 72 °C/ 1.5 min (35 cycles), final extension, 72 °C/10 min (one cycle), then kept at 4 °C until use. Agarose gel (1.5%) electrophoresis was used for separating the PCR products. The run was performed at 100 V for about 1 h. The DNA marker used in this study was 100 bp DNA ladder.

**Table 3:** List of SCoT primers, sequences.

Primer	Name	Sequence	Primer	Name	Sequence
1	SCoT-01	CAACATGGCTACCACCA	6	SCoT-18	ACCATGGCTACCACCGCC
2	SCoT-03	CAACAATGGCTACCACCG	7	SCoT-19	ACCATGGCTACCACCGGC
3	SCoT-05	CAACAATGGCTACCACGA	8	SCoT-21	ACGACATGGCGACCCACA
4	SCoT-13	ACGACATGGCGACCATCG	9	SCoT-28	CCATGGCTACCACCGCCA
5	SCoT-15	ACGACATGGCGACCGCGA	10	SCoT-34	ACCATGGCTACCACCGCA

### Gel images and bioinformatic analysis

The resulting gel images were examined using GelAnalyzer3 software to determine the molecular sizes of the amplified fragments. The amplified fragments were scored as present (1) or absent (0). Polymorphic Information Content (PIC), Expected Heterozygosity (He), and to determine the Effective Multiplex Ratio (EMR) values, the online software (https://irscope.shinyapps.io/iMEC/) was utilized^46^. The resulting data was then used to create a dendrogram of similarities and relationships between new sesame lines using the NTSYS program^47^. For bioinformatic analysis, SCoT results and accessions on databases were compared using BLAST multiple alignment techniques (NCBI) (BLAST: Basic Local Alignment Search Tool) to catch regions that contain annotation of genes related to SCoT sequences through gene portal (https://www.ncbi.nlm.nih.gov/gene/), 3D structure of proteins obtained using UniProt portal (https://www.uniprot.org/) and protein analysis found on Expasy protparam portal (https://web.expasy.org/protparam/).

## Results

### Mean performance and variance

**Fig. 1:**
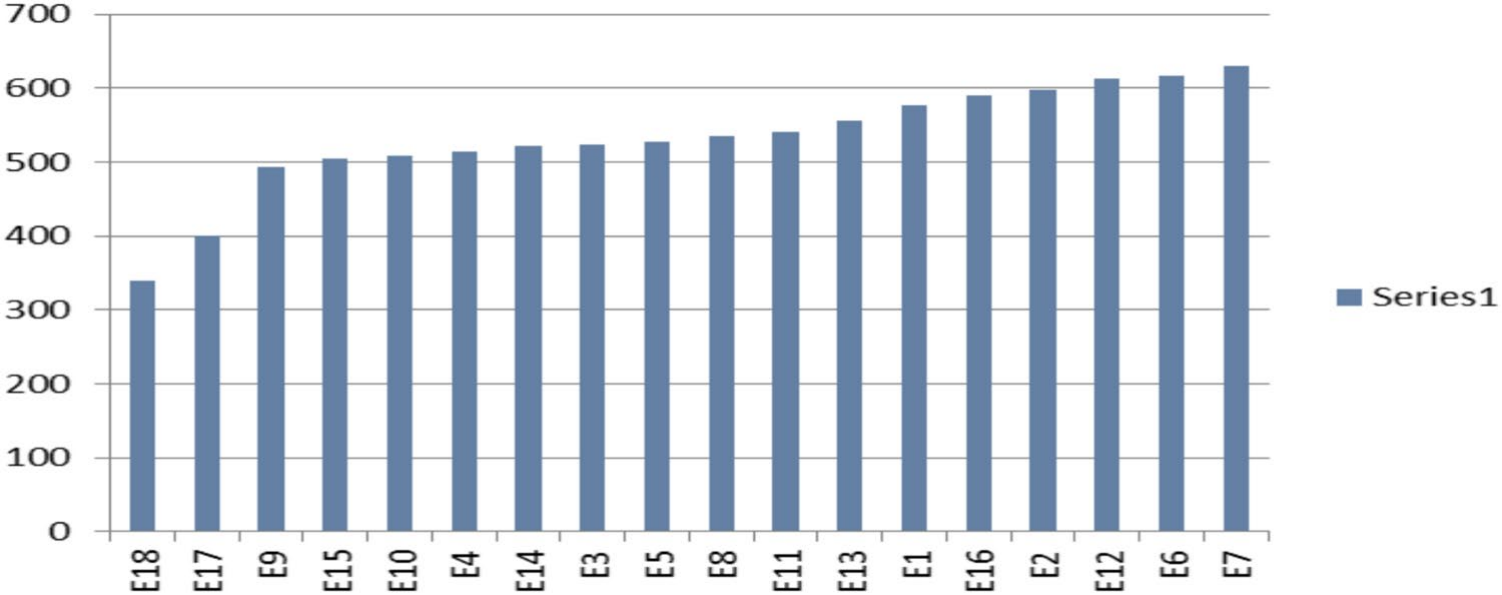
Showed the mean performance environments for seed yield under drought stress.

Table 4 showed that nine lines (C1-5, C2-6, C3-8, C5-8, C6-3, C6-9, C6-11, C9-3, and C9-20) outperformed commercial varieties in seed yield across 18 environments under drought conditions, with percentages ranged from 2.0 to 32.0%. The line C5.8 produced the highest seed yield (643.3 kg ha-1), followed by line C6.9 (638.5 kg ha-1). Fourteen lines (C2-2, C5-7, C6-2, C6-3, C6-11, C6-12, C8-11, C9-20, C3-8, C9-6, C9-3, C2-6, C6-9, and C5-8) produced more seeds than commercial varieties under full irrigation (N). In terms of environments, the environments showed a clear difference in seed production, with environment No. 6 recording the highest average yield, followed by environment 11, then environments 12, 2, and 7 (Fig. 1).

**Table 4:** Seed yield ha^-1^ and variances of sesame genotypes under drought conditions.

Site	Beni Suef (Northern Central Egypt)									Giza (Northern Egypt)						Nubaria (western delta of Egypt)					$\overline{X }$	$\overline{\overline{X}}$	N
Envi	E1	E2	E3	E4	E5	E6	E7	E8	$\overline{X }$	E9	E10	E11	E12	E13	$\overline{X }$	E14	E15	E16	E17	E18			
Gen.\year	2020		2021		2022		2023			2020		2021		2022		2020	2021	2023	2023				
C1	600.4	706.4	522.3	562.6	664.0	638.3	615.7	541.9	574.1	602.9	544.3	637.6	730.5	601.1	657.5	634.2	377.8	356.7	282.0	337.2	340.4	525.8	1005.0
C2	608.8	682.8	525.1	526.5	617.9	630.0	598.5	535.8	553.3	586.5	499.4	631.8	525.7	541.0	582.3	556.0	332.5	314.0	248.2	296.7	299.6	480.7	920.0
C1-5	675.2	659.4	586.2	638.6	649.1	658.4	644.5	564.6	549.9	625.1	530.6	634.3	643.9	781.7	558.8	629.9	375.9	304.9	370.0	343.1	351.8	535.6	963.0
C1-6	633.2	607.0	507.6	633.2	647.8	661.4	615.0	558.0	622.3	609.5	571.2	659.4	502.4	510.9	633.8	575.5	304.5	425.0	305.0	348.3	337.4	507.5	970.0
C1-8	582.6	626.3	494.0	516.6	588.5	585.5	565.6	557.4	524.7	560.1	531.9	741.0	583.0	497.3	599.7	590.6	221.9	234.5	261.7	363.0	277.4	476.0	904.0
C1-9	674.3	739.2	660.8	680.1	743.4	798.6	716.1	696.2	525.6	692.7	478.8	467.3	457.2	448.0	481.0	466.5	245.6	340.0	244.0	278.6	270.4	476.5	678.0
C2-2	586.9	610.6	538.7	493.8	623.4	618.2	578.6	577.7	763.1	599.0	607.2	762.0	607.0	408.3	563.2	589.5	305.2	276.2	305.9	350.3	316.9	501.8	1069.0
C2-3	422.4	472.7	443.5	377.8	470.6	470.6	442.9	413.1	364.7	430.9	485.7	560.6	615.4	388.8	450.4	500.2	310.7	323.4	315.4	365.7	327.8	419.6	644.0
C2-6	442.9	447.9	364.1	391.9	431.3	487.7	427.6	440.6	445.1	431.0	596.7	715.9	734.9	976.6	875.9	780.0	429.6	542.7	599.9	572.6	540.9	584.0	1133.0
C3-4	387.0	480.6	519.7	507.5	507.5	529.0	488.6	509.2	366.8	477.3	358.0	407.0	474.7	403.2	427.5	414.1	306.0	265.4	193.5	224.9	248.3	379.9	701.0
C3-7	513.1	609.4	659.5	654.8	698.8	698.3	639.0	630.7	510.3	623.8	508.0	634.3	523.4	498.2	516.3	536.0	246.7	324.3	256.4	362.2	318.0	492.6	953.0
C3-8	528.6	580.8	659.4	716.0	705.0	693.9	647.3	631.3	644.2	645.2	751.5	634.3	628.2	864.6	805.9	736.9	390.2	356.2	430.9	385.3	387.8	590.0	1080.0
C5-7	523.4	533.3	497.3	695.9	690.8	630.6	595.2	549.6	565.7	586.9	521.4	715.9	556.7	355.9	443.9	518.8	318.3	426.7	459.1	358.8	382.9	496.2	1010.0
C5-8	661.2	585.5	586.2	638.6	727.5	670.5	644.9	584.3	534.4	625.9	681.4	772.6	887.5	806.1	904.2	810.4	488.2	482.0	483.6	499.7	493.6	643.3	1108.0
C6-2	641.7	607.0	533.8	596.5	620.6	684.4	614.0	581.6	642.8	613.6	302.3	405.3	623.8	415.7	374.8	424.4	332.7	435.2	448.6	407.9	412.8	483.6	978.0
C6-3	622.9	596.7	570.5	491.9	509.8	579.0	561.8	544.7	515.3	554.7	652.2	791.4	728.3	537.1	735.8	689.0	489.4	557.0	565.3	604.7	554.0	599.2	1058.0
C6-4	674.0	635.9	598.1	533.8	512.6	638.9	598.9	518.8	538.0	583.2	483.6	544.3	506.5	424.0	536.9	499.1	326.1	306.8	277.5	271.7	296.1	459.5	912.0
C6-5	429.6	502.7	442.1	423.8	448.7	569.8	469.5	524.2	551.4	484.6	437.6	431.2	397.8	498.2	468.9	446.7	340.4	321.9	284.7	293.9	311.0	414.1	883.0
C6-7	429.3	487.7	486.9	427.2	448.1	522.7	467.0	555.0	367.9	465.8	615.5	641.7	575.0	430.2	524.9	557.5	329.5	336.9	330.1	430.4	371.4	464.9	889.0
C6-9	605.3	654.4	752.1	712.0	665.7	714.2	684.0	660.6	496.5	660.5	727.3	718.2	722.5	746.9	736.2	730.2	549.4	606.6	450.5	522.4	524.6	638.5	1118.0
C6-11	647.8	685.6	749.4	785.6	715.2	806.5	731.7	688.9	730.3	726.8	791.2	697.0	796.7	685.6	687.3	731.6	446.7	360.4	391.8	477.3	420.3	626.2	1101.0
C6-12	507.8	554.9	555.8	571.2	542.5	621.6	559.0	579.2	629.8	569.1	505.7	615.4	486.8	654.0	633.0	579.0	375.6	436.5	397.5	423.5	409.7	519.2	986.0
C8-4	627.9	623.5	558.8	589.5	631.4	612.7	607.3	552.4	446.6	583.3	453.7	555.2	513.0	437.6	516.0	495.1	210.1	268.0	276.9	266.1	254.6	444.3	553.0
C8-8	649.9	613.2	529.6	612.5	645.2	611.0	610.2	485.3	643.8	600.1	526.6	676.1	433.3	518.1	708.2	572.5	395.0	419.8	435.9	343.9	389.0	520.5	946.0
C8-11	647.5	688.7	592.2	702.2	716.4	703.0	675.0	589.9	488.0	644.8	503.7	596.7	647.8	409.3	473.4	526.2	331.7	352.7	297.3	355.1	326.4	499.1	1023.0
C9-3	783.8	788.6	785.4	804.2	803.8	784.3	791.7	755.9	705.9	778.2	617.7	797.6	661.6	557.7	724.5	671.8	388.2	400.7	363.7	412.2	384.0	611.3	1162.0
C9-6	678.9	666.9	685.7	678.2	733.4	650.5	682.3	650.5	742.0	685.4	337.1	504.6	547.8	394.5	395.9	436.0	417.9	388.2	356.7	312.9	361.0	494.1	1206.0
C9-7	487.7	489.3	494.4	455.2	518.0	509.8	492.4	447.1	545.3	493.2	548.6	641.9	463.8	455.8	511.4	524.3	437.1	434.9	514.3	461.6	461.3	492.9	948.0
C9-15	457.5	546.5	527.1	467.3	562.8	546.8	518.0	474.6	385.9	498.5	508.7	565.3	591.7	492.0	535.2	538.6	333.3	296.1	296.1	303.2	308.8	448.6	799.0
C9-20	598.5	544.9	615.6	547.5	635.6	597.2	589.9	576.4	539.1	582.7	551.5	763.1	837.4	825.7	657.2	727.0	475.2	567.1	579.5	487.7	518.0	609.2	1082.0
$\overline{X }$	577.7	600.9	568.1	581.1	615.8	630.8	595.7	565.9	550.4	587.4	541.0	630.6	600.1	552.1	590.7	582.9	361.0	382.0	367.4	382.0	373.2	514.5	959.4
SL0.05	**	**	**	**	**	**	**	**	-	**	**	**	**	**	-	**	**	**	**	**	-	-	**
LSD 0.05	108	87	92	106	107	96	99	90	-	50	56	65	69	77	-	34	34	38	32	35.8	-	-	151.8
CV%	15.9	12.3	12.8	15.5	14.2	13	13.9	13.5	-	8	7.6	9	10	11	-	8	8	9	7	8.2	-	-	17.5

E: represent environments, $\overline{\text{X} }$: overall mean under drought stress,$\overline{\overline{\text{X}}}$ : overall mean under full drouight, conditions N: overall mean under full irrigation conditions, **significant level at p ≤ 0.05, *LSD* least significant different, *CV%* coefficient of variation.

**Fig. 2:**
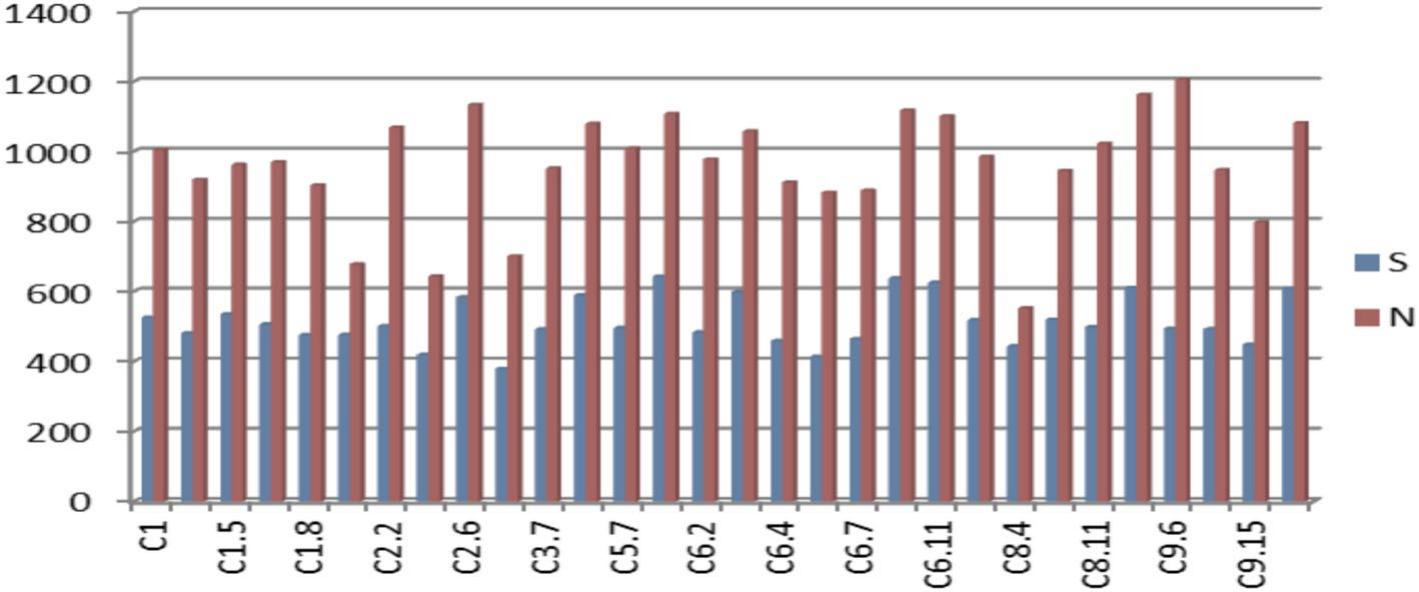
Behavior of sesame genotypes in relation to seed yield under drought (S) and natural conditions (N).

We found that four lines (C2-2, C9-6, C6-11, and C3-8) produced the greatest amount of seed compared to the other genotypes under drought stress in the Beni Suef site in north-central Egypt. At the Giza site in northern Egypt, we found three lines (C5-8, C2-6, and C3-8) produced the greatest seed yield. At the Nubaria site in Egypt’s western delta, we found that seven lines (C6-3, C6-9, C9-7, C5-8, C2-6, C6-11, and C3-8) produced the highest seed yield. Additionally, regarding productivity, seven lines (C2-6, C5-8, C6-3, C6-9, C6-11, C9-3, and C9-20) outperformed the control in natural and drought conditions (Fig. 2). Additionally, genotypes showed significant variations (p < 0.05) in seed yield across environments.

### Combine analysis

We performed a combined pooled analysis of variance because there were significant differences between the genotypes in each environment in which they were grown. Significant differences (p ≤ 0.05) in seed production were observed due to the combined effect of environment and genotype as well as the GEI, providing chances for evaluation and selection (Table 5).

**Table 5:** Combined pooled analysis of variance for seed yield obtained from trials conducted under drought conditions.

Source of variation	DF	SS	MS
Replications	3	210,443.0	70,148.0*
Genotypes (G)	29	9,059,728.0	312,404.0*
Environments (E)	17	1,095,138.0	644,199.0*
G*E	493	11,834,081.0	24,004.0*
Residual	1617	29,859,288.0	18,466.0*
Total	2159	61,914,927.0	
CV%	13.2		

*DF* Degree of freedom, *SS* sum of squares, *MS* mean square. *significant level at p ≤ 0.05**,** *CV%* coefficient of variation.

### MMI and principal component analysis (PCA)

Given the significant relationship between genotypes and environments, which revealed that genotype performance varies across environments, we utilized AMMI and GGE biplot models to predict genotype adaptation and stability across diverse environments. The AMMI used traditional ANOVA to analyze the main effects (additive part) of genotypes and environment, as well as the principal components of the analysis of genotype-by-environment interaction. The results of AMMI analysis shown in Table 6 showed significant differences among genotypes, environments, interactions, and IPC1 fragments. Since the combined contribution of IPC1 and IPC6 justified 92.0% of the interaction, the AMMI model seemed acceptable in estimating the interaction. In Table 7, the top four lines that performed best across environments and were selected based on the AMMI model are shown. Line C6-11 was selected in 13 environments, followed by C9-3 in 11 environments, and two lines (C6-9 and C5-8) in 9 environments.

**Table 6:** NOVA Table for AMMI model.

Source of variation	df	SS	MS
Treatments	539	31,845,195	59,082 **
Genotypes	29	9,059,728	312,404**
Environments	17	10,951,386	644,199**
Block	54	22,027,340	407,914**
Interactions	493	11,834,081	24,004**
IPCA 1	45	6,444,354	143,208**
IPCA2	43	1,214,043	28,234**
IPCA3	41	1,067,050	26,026**
IPCA4	39	936,078	24,002**
IPCA5	37	705,891	19,078**
IPCA6	35	534,537	15,272**
Residuals	253	932,129	3684
Error	1566	8,042,391	5136
Total	2159	61,914,927	28,678
CV%	13.2		

*DF* Degree of freedom, *SS* sum of squares, *MS* mean square. **significant level at p ≤ 0.05, *CV%*, coefficient of variation.

**Table 7:** Showed the first four AMMI selections for each environment.

Environment	1	2	3	4
E6	C9-3	C6-11	C1-9	C8-11
E2	C9-3	C9-6	C1-9	C6-11
E5	C9-3	C1-9	C6-9	C6-11
E7	C9-3	C6-11	C1-9	C9-6
E1	C9-3	C9-6	C1-9	C8-8
E4	C6-11	C9-3	C9-6	C6-9
E3	C9-3	C6-11	C6-9	C1-9
E10	C2-2	C9-6	C6-11	C9-3
E8	C9-3	C6-11	C6-9	C1-9
E9	C6-11	C9-3	C9-6	C3-8
E18	C6-9	C6-3	C9-20	C5-8
E13	C5-8	C6-9	C6-3	C6-11
E17	C6-9	C6-3	C2-6	C5-8
E12	C6-3	C5-8	C6-11	C9-3
E11	C6-11	C3-8	C6-9	C5-8
E14	C6-9	C5-8	C2-6	C6-11
E16	C2-6	C5-8	C3-8	C6-9
E15	C2-6	C3-8	C5-8	C9-20

### GGE biplot

**Fig. 3:**
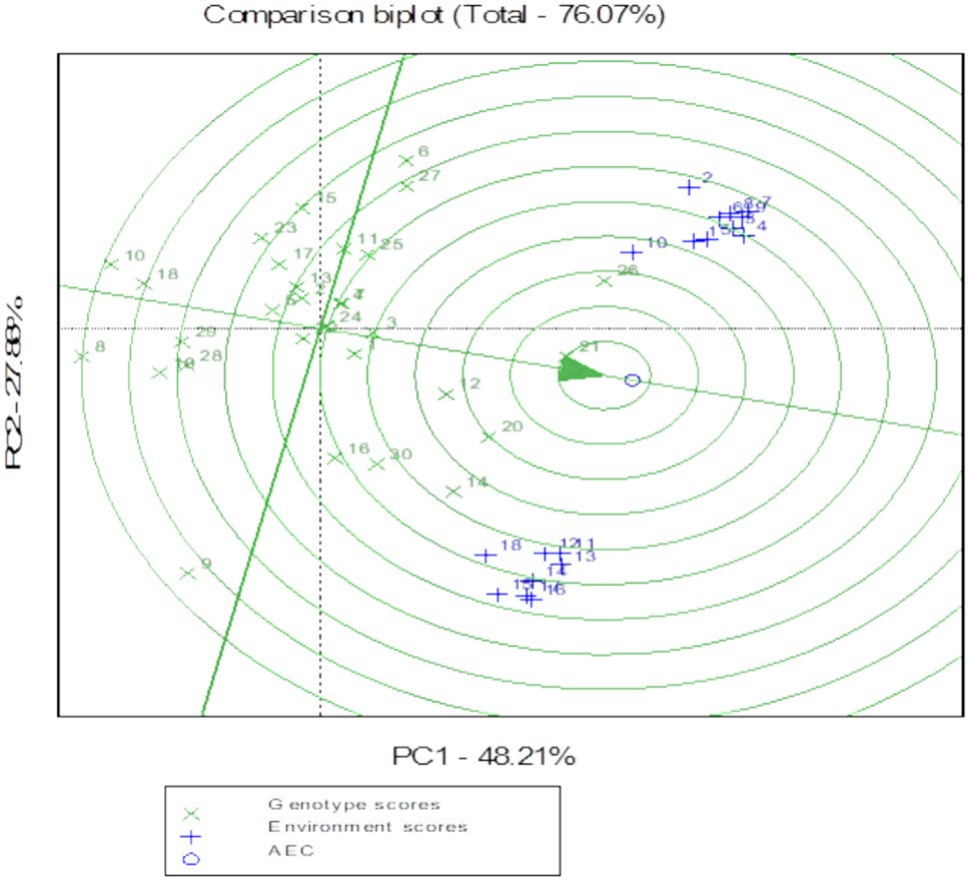
Comparison of genotypes based on PC1 and PC2 showing G × E interactions of the 30 sesame genotypes under drought conditions.

The GGE biplot model is useful for understanding the effects of GEI and environmental adaptable genotypes identification. As illustrated in Fig. 3, the optimal genotype is invariably found in the center and towards the top of the arrow within the circular band, and the inner circle contained C6-11, followed by C3-8, C6-9, C9-3, C5-8, and C1-5. In contrast, it was observed that C3-4, C2-3, and C6-2 showed the maximum separation from the plot’s arrowhead.

### Parametric and non-parametric statistics

Parametric and non-parametric statistics were ranked the genotypes based on seed yield under drought stress (Table 8), line C6-11, which was ranked first in seed production, was selected on the basis of one parametric statistics (SD) and sex non-parametric statistics (S^(1)^, S^(2)^, S^(3)^, S^(6)^, NP^(2)^ and NP^(4)^). Line C6-9, which ranked second in seed production, had been selected based on two parametric statistics (CVi and SD) and four non-parametric statistics [S^(3)^, S^(6)^, NP^(3)^, and NP^(4)^]. Line C5-8, ranked third in seed production, was selected based on one parametric statistic (SD) and four non-parametric statistics [S^(3)^, S^(6)^, NP^(3)^, and NP^(4)^]. Line C9-3 was ranked fourth in seed production and selected using five non-parametric statistics [S^(2)^, S^(3)^, NP^(2)^, NP^(3)^, and NP^(4)^].

**Table 8:** Ranks sesame genotypes according to seed yield and parametric and nonparametric statistics under drought circumstances.

Genotype	Y	Parametric statistics			Non-parametric statistics								SD
		W^i2^	σ^2i^	CVi	S⁽^1^⁾	S⁽^2^⁾	S⁽^3^⁾	S⁽⁶⁾	NP⁽^1^⁾	NP⁽^2^⁾	NP⁽^3^⁾	NP⁽^4^⁾	
C1	10.0	5.0	5.0	17.0	12.0	10.0	9.0	7.0	5.0	4.0	7.0	8.0	3.7
C2	21.0	2.0	2.0	19.0	9.0	9.0	13.0	13.0	2.0	14.0	10.0	13.0	6.3
C1.5	9.0	12.0	12.0	16.0	8.0	6.0	5.0	5.0	3.0	6.0	3.0	6.0	4.0
C1.6	14.0	4.0	4.0	15.0	11.0	11.0	11.0	11.0	8.0	2.0	8.0	9.0	4.0
C1.8	23.0	10.0	10.0	28.0	18.0	17.0	18.0	19.0	9.0	20.0	21.0	20.0	5.7
C1.9	18.0	29.0	29.0	30.0	29.0	29.0	29.0	28.0	29.0	27.0	27.0	29.0	3.2
C2.2	16.0	20.0	20.0	25.0	19.0	20.0	16.0	14.0	7.0	10.0	13.0	15.0	4.9
C2.3	29.0	11.0	11.0	6.0	10.0	12.0	26.0	29.0	17.0	28.0	29.0	30.0	9.4
C2.6	8.0	30.0	30.0	27.0	30.0	30.0	30.0	30.0	30.0	21.0	26.0	28.0	6.5
C3.4	30.0	3.0	3.0	22.0	1.0	1.0	7.0	23.0	5.0	30.0	30.0	23.0	12.4
C3.7	17.0	13.0	13.0	23.0	20.0	19.0	15.0	15.0	21.0	12.0	18.0	16.0	3.5
C3.8	6.0	23.0	23.0	18.0	13.0	14.0	8.0	6.0	20.0	16.0	6.0	5.0	6.9
C5.7	19.0	19.0	19.0	13.0	22.0	22.0	19.0	17.0	12.0	13.0	17.0	18.0	3.3
C5.8	3.0	24.0	24.0	9.0	7.0	7.0	3.0	4.0	19.0	7.0	4.0	4.0	8.0
C6.2	20.0	27.0	27.0	14.0	24.0	25.0	24.0	18.0	16.0	11.0	19.0	18.0	5.2
C6.3	7.0	26.0	26.0	2.0	26.0	27.0	22.0	16.0	28.0	8.0	15.0	14.0	9.0
C6.4	24.0	7.0	7.0	20.0	16.0	18.0	21.0	21.0	4.0	26.0	20.0	21.0	7.2
C6.5	28.0	8.0	8.0	8.0	5.0	4.0	17.0	25.0	14.0	29.0	28.0	26.0	10.0
C6.7	26.0	18.0	18.0	7.0	23.0	23.0	25.0	26.0	22.0	23.0	25.0	27.0	5.5
C6.9	2.0	15.0	15.0	3.0	4.0	8.0	4.0	2.0	18.0	9.0	2.0	2.0	5.9
C6.11	1.0	16.0	16.0	12.0	2.0	2.0	1.0	1.0	15.0	17.0	1.0	1.0	7.3
C6.12	12.0	6.0	6.0	4.0	14.0	13.0	10.0	10.0	13.0	5.0	11.0	10.0	3.4
C8.4	25.0	9.0	9.0	29.0	15.0	15.0	20.0	24.0	10.0	22.0	24.0	24.0	7.0
C8.8	11.0	14.0	14.0	10.0	17.0	16.0	14.0	9.0	10.0	3.0	9.0	11.0	3.8
C8.11	15.0	17.0	17.0	24.0	25.0	24.0	23.0	20.0	23.0	19.0	16.0	17.0	3.6
C9.3	4.0	21.0	21.0	21.0	6.0	5.0	2.0	3.0	26.0	1.0	5.0	3.0	9.4
C9.6	13.0	28.0	28.0	26.0	28.0	28.0	27.0	22.0	27.0	18.0	22.0	22.0	4.8
C9.7	22.0	22.0	22.0	1.0	27.0	26.0	28.0	27.0	24.0	24.0	23.0	25.0	7.1
C9.15	27.0	1.0	1.0	11.0	3.0	3.0	6.0	12.0	1.0	25.0	14.0	12.0	9.0
C9.20	5.0	25.0	25.0	5.0	21.0	21.0	12.0	8.0	25.0	15.0	12.0	7.0	8.0

Y: seed yield, Parametric statistics included: W^2i^: the covalence of the genotype as measured by its interaction with the environment, squared and summed across environments. σ^2i^: the stability variance of genotype across environments after the main effects of environmental factors have been removed. CVi: the combined values of the coefficient of variation, average yield, and environmental variance. Non-parametric statistics included: S (1): the mean of a genotype’s absolute rank differences across all tested environments; S (2): the variance among ranks across all tested environments; S (3): the sum of absolute deviations for each genotype relative to the mean of ranks; S (6): the sum of rank squares for each genotype relative to the mean of ranks. Four NP (1–4) statistics are a set of alternative non-parametric stability statistics are based on the ranks of adjusted means of the genotypes in each environment. Four NP (1–4) statistics are a group of alternative non-parametric stability statistics based on the genotypes’ adjusted mean ranks in each environment. *SD* stander deviation.

### Correlation coefficients

Table 9 showed correlation coefficients between the yield and the parametric and non-parametric statistics under drought conditions. Yield has positive correlation significantly with S⁽^3^⁾, S⁽^6^⁾, NP⁽^2^⁾, NP^⁽3⁾^, and NP^⁽4⁾^, in contrast, was significantly negatively correlated with W_i_^2^, σ^2^_i_ and NP⁽^1^⁾. W_i_^2^ and σ^2i^ were significantly positively correlated with σ^2^_i_, S⁽^1^⁾, S⁽^2^⁾, S⁽3⁾, and NP⁽^1^⁾. S⁽^1^⁾ was significantly positively correlated with S⁽^2^⁾, S⁽3⁾, S⁽^6^⁾, NP⁽^1^⁾, NP^⁽3⁾^, NP^⁽4⁾^, but was significantly negatively correlated with SD. S^⁽2⁾^ has significant positive correlation with S⁽^3^⁾, S^⁽6⁾^, NP⁽^1^⁾, NP⁽^3^⁾ and NP^⁽4⁾^ and was significantly negatively correlated with SD. S^⁽3⁾^ was significantly positively correlated with S^⁽6⁾^, NP⁽^1^⁾, NP^⁽2⁾^, NP⁽^3^⁾ and NP^⁽4⁾^. NP^⁽2⁾^ was significantly positively correlated with NP⁽^3^⁾, NP⁽4⁾ and SD. NP⁽^3^⁾ was significantly positively correlated with NP^⁽4⁾^.

**Table 9:** Simple correlation between Y and parametric and non-parametric statistics under drought conditions.

	W_i_^2^	σ^2^_i_	CVi	S⁽^1^⁾	S⁽^2^⁾	S⁽^3^⁾	S⁽⁶⁾	NP⁽^1^⁾	NP⁽^2^⁾	NP⁽^3^⁾	NP⁽4⁾	SD
Y	− 0.454	− 0.454**	0.147	0.023	0.02	0.451**	0.725**	− 0.370**	0.690**	0.774**	0.759**	0.215
W_i_^2^		0.999**	0.041	0.652**	0.673**	0.402**	0.12	0.827**	− 0.064	0.059	0.087	− 0.119
σ^2^_i_			0.041	0.652**	0.673**	0.402**	0.12	0.827**	− 0.064	0.059	0.087	− 0.119
CVi				0.990**	0.830**	0.536**	0.584**	0.088	0.421**	0.500**	− 0.467**	− 0.224
S⁽^1^⁾					0.990**	0.830**	0.536**	0.584**	0.088	0.421**	0.500**	− 0.467**
S⁽2⁾						0.842**	0.540**	0.596**	0.113	0.424**	0.503**	− 0.433**
S⁽3⁾							0.877**	0.427**	0.479**	0.770**	0.856**	− 0.198
S⁽6⁾								0.057	0.196	0.185	0.002	0.108
NP⁽^1^⁾									0.057	0.196	0.185	0.002
NP⁽2⁾										0.783**	0.742**	0.435**
NP⁽3⁾											0.968**	0.236
NP⁽4⁾												0.088

**: significant level at 0.05.

### Genetic Marker SCoT-PCR analysis

**Fig. 4:**
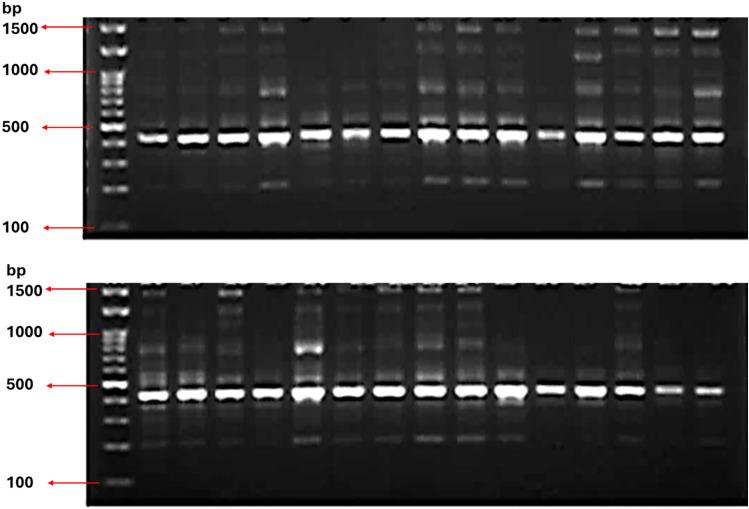
PCR amplification profiles of 30 sesame genotypes obtained with Scot-21 primer, M: 100 bp ladder.

Ten SCoT primers were used for amplification among 30 sesame genotypes. Figure 4 illustrates an example of SCoT-PCR marker profile created by primer SCoT-21. The 10 primers produced a total number of 106 fragments, with an average of 10.6 fragments per primer ranging from 7 to17 fragments with product size ranged from 120 to 2500 bp. The polymorphism percentages ranged from 44.44% with primer SCoT-01 to 100.00% primer SCoT-13 with an average of 75.31% (Table 10). SCoT-13’s ability to detect 100% polymorphic bands in wheat germplasm enables precise differentiation of genotypes, even in closely related cultivars^48^. The average of PIC was 0.277 (has range from 0.087 to 0.358), which reflects a reasonable percentage of diversity between new lines. As Known that PIC ranged from 0 to 1 and a higher PIC value indicates a greater level of polymorphism and diversity among the genotypes being studied. Also, Primer SCoT-28 reveals the highest degree 0.64 of the expected heterozygosity. SCoT-21 has 88.24%, 0.3580, and 10.03 for P %, PIC and EMR% respectively. In other experiment among sex local sesame cultivars in Saudi Arabia the highest of P %, PIC, MI, and EMR%, were recorded with primer SCoT-21^49^.

**Table 10:** Presents the results of SCoT-PCR amplification and polymorphism.

Primer no	Primer name	Band size bp	Total Bands	P bands	P %	PIC	EMR	He
1	SCoT-01	350–2500	9	4	44.44	0.1897	7.03	0.21
2	SCoT-03	400–1990	8	5	62.5	0.087	6.69	0.08
3	SCoT-05	200–1100	8	7	87.5	0.3144	5.17	0.39
4	SCoT-13	250–700	7	7	100	0.299	4.55	0.37
5	SCoT-15	220–1800	9	5	55.56	0.34	5.45	0.43
6	SCoT-18	230–1700	14	10	71.43	0.299	4.55	0.37
7	SCoT-19	240–830	8	7	87.5	0.339	4.79	0.43
8	SCoT-21	200–1560	17	15	88.24	0.358	10.03	0.47
9	SCoT-28	120–1250	14	9	64.29	0.287	1.09	0.64
10	SCoT-34	250–1550	12	11	91.67	0.256	8.97	0.3
Total			106	80				
Average			10.6	8	75.31	0.277	5.83	

*PIC* Polymorphic information content, *EMR* Effective multiplex ratio, *He* expected heterozygosity.

### Bioinformatics analysis

**Fig. 5:**
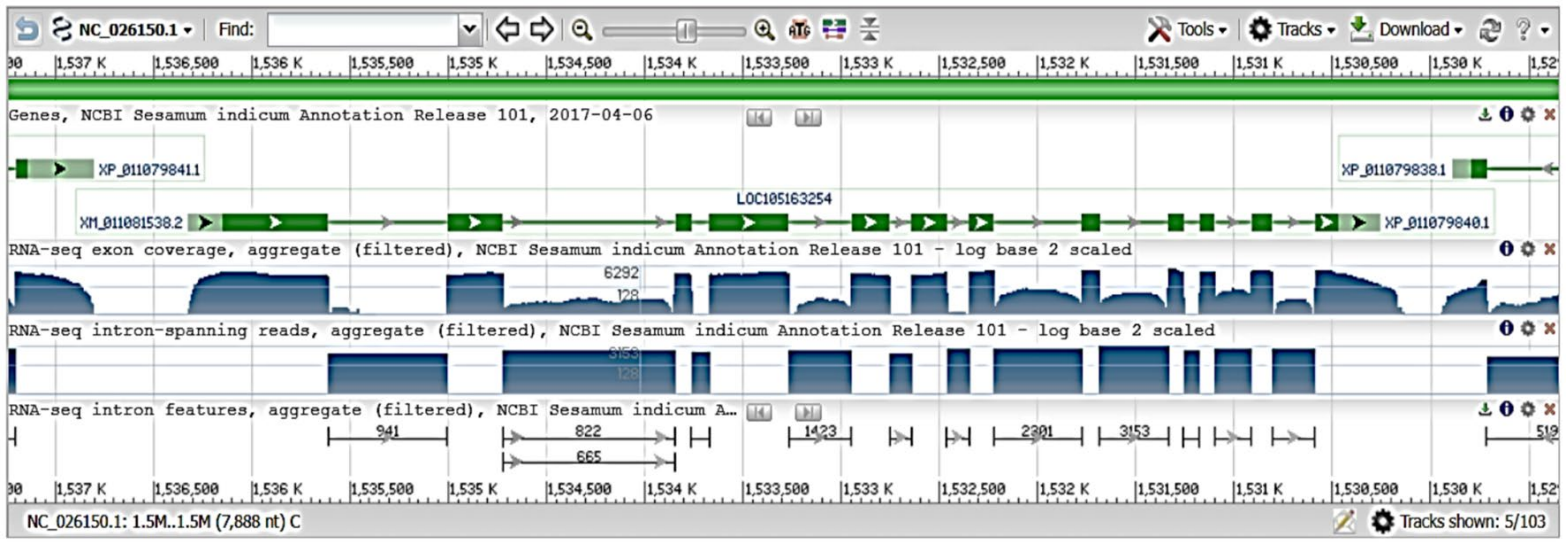
Annotations and positions of DNA repair helicase gene in *Sesamum indicum* (LOC105163254).

According to GenBank and multiple alignment techniques, SCoT-21 catches a region that contains a sequence that may encode DNA repair helicase XPD gene (Fig. 5) in *Sesamum indicum* (LOC105163254).

**Fig. 6:**
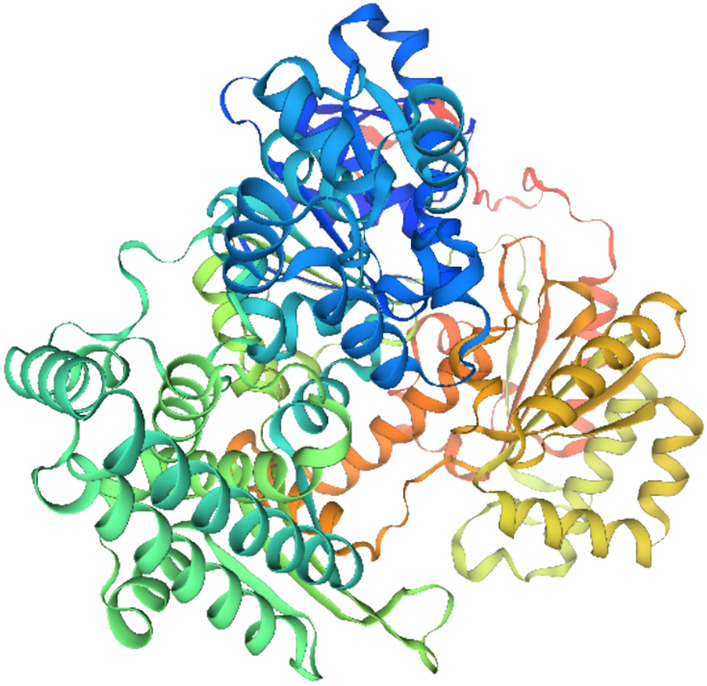
3D structure of protein related to DNA repair helicase XPD gene.

The DNA repair helicase XPD gene contains 6068 bp and encodes a protein of 757 aa. DEAD/DEAH box DNA helicases are a type of enzyme that utilizes energy from ATP to unwind the DNA double helix, a process essential for various cellular activities like DNA replication, repair, and transcription (Brosh, 2013). The 3D structure of the protein revealed that it contains two basic domains **(**Fig. 6). The helicases are characterized by a specific amino acid sequence motif called the DEAD/DEAH box, named after the single-letter codes of the amino acids Asp-Glu-Ala-Asp/His^50^. As Known that PIC ranged from 0 to 1 and a higher PIC value indicates a greater level of polymorphism and diversity among the genotypes being studied. Also, Primer SCoT-28 reveals the highest degree 0.64 of the expected heterozygosity.

**Fig. 7:**
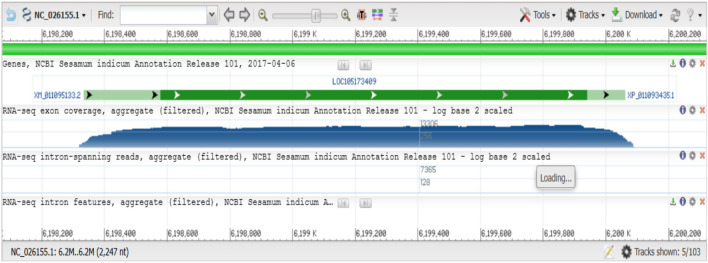
Annotations and positions of malonyl-coenzyme: anthocyanin 5-O-glucoside-6′′′-O-malonyl transferase-like gene in *Sesamum indicum* (LOC105173409).

**Fig. 8:**
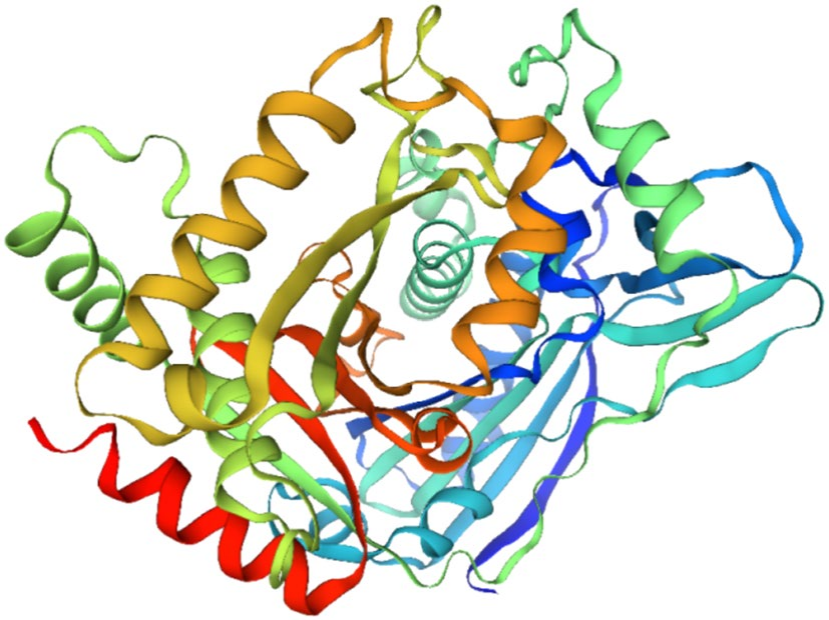
3D structure of protein related to DNA repair helicase XPD gene.

According to GenBank and multiple alignment techniques, SCoT-28 catches a region that contains a sequence that may encode malonyl-coenzyme: anthocyanin 5-O-glucoside-6′′′-O-malonyl transferase-like gene (Fig. 7) in *Sesamum indicum* (LOC105173409). In sesame, the enzyme malonyl-coenzyme A: anthocyanin 5-O-glucoside-6′′′-O-malonyltransferase-like is involved in the modification of anthocyanins, the pigments responsible for the vibrant colors of many flowers and fruits. This enzyme catalyzes the transfer of a malonyl group from malonyl-CoA to the 6′′′-hydroxyl group of the 5-O-glucoside moiety of anthocyanins. This modification is important for stabilizing anthocyanins and altering their color intensity^51,52^. The enzyme belongs to the BAHD acyltransferase family, a diverse group of enzymes involved in the acylation of various plant secondary metabolites^53^. The 3D structure of the protein revealed that it contains two basic domains (Fig. 8).

**Fig. 9:**
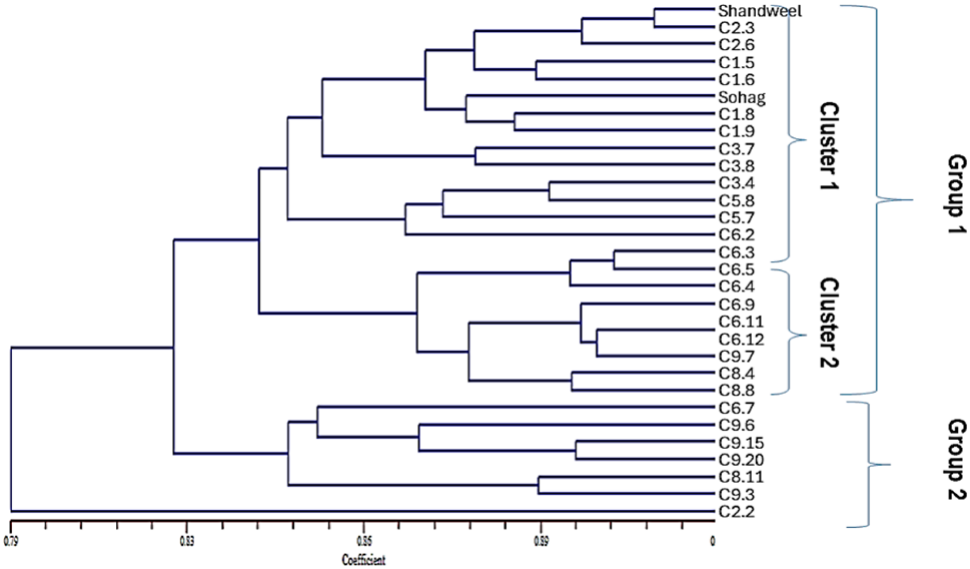
Phylogenetic tree of 28 new lines sesame and two varieties based on the similarity’s distances coefficient.

Based on the 0–1 data of SCoT-PCR markers, NTSYS program was used to measure the similarities between the new sesame lines. According to the phylogeny tree, lines of sesame are divided into two groups, II and the outer group line C2-2 which is less related to the other lines as shown in Fig. 9. Group I consist of two big clusters; Cluster I contain (Shandweel, C2-3, C2-6, C1-5, C1-6, Sohag, C1-8, C1-9, C3-7, C3-8, C3-4, C5-8, C5-7 and C6-2) whereas Cluster II includes (C6-3, C6-5, C6-4, C6-9, C6-11, C6-12, C9-7, C8-4 and C8-8). On the other side group II contain (C6-7, C9-6, C9-15, C9-20, C8-11 and C9-3).

## Discussion

Climate change has led to the effects of drought on agricultural productivity being more severe than they were previously, necessitating the development of several strategies for reducing the problem^54,55^. A reasonable and natural approach to solving this problem is to breed field crops to resist drought^56,57^. Discovering genetic diversity across different genetic resources is an essential phase in developing drought-tolerant/resistant^58^. In this study we evaluated the performance of sesame genotypes (28 new lines) for seed production under normal and drought conditions at three locations over four years, creating 18 environments to identify high-yielding and more stable lines to increase the supply of commercial varieties available within Egypt to mitigate the negative effects of climate change, which represents horizontal resistance. References^59,60^ suggested that increasing adaptive genetic variation reduces the adverse effects of climate change. Based on LSD values, we found that nine lines (C1-5, C2-6, C3-8, C5-8, C6-3, C6-9, C6-11, C9-3, and C9-20) performed better in terms of seed yield than the control (C1: excellent variety) (Table 4). The high seed yield may have been a result of these lines responding favorably to their growing conditions and carrying genes that assist in drought tolerance. This is because increased variation raises the possibility of discovering novel genotypes that are more resistant to climate change. Furthermore, according to^61^, these lines have longer rooted than the control, which aids in their ability to absorb water and perform important plant functions under drought stress compared to control. Reference^62^ suggested that lateral roots and tiny roots may be an adaptation mechanism for enhancing water intake during drought stress by offering a more absorbent surface. In this study, significant variation (p ≤ 0.05) emerged among genotypes for seed yield across environments^3,13,63,64^, which may be due to different environmental conditions, as shown in Table 1 ref.^65^ and different soil properties in the experimental sites^66,67^. Additionally, these lines may demonstrate various gene expression pathways that regulate seed yield^25,68^. The present study helped us to identify the genetic resources suitable for drought and optimal conditions of clay soil (C2-2, C9-6, C6-11, C9-3, C5-8, and C2-6) and sandy soil (C6-3, C9-7, C6-11, C9-3, C6-9, C5-8, C9-20, and C2-6), and identify the lines that recorded the highest productivity under different conditions, namely: C6-11, C5-8, and C2-6^69^. Therefore, the current study suggested the establishment of targeted or site-specific hybridization programs, and this was linked to the fact that these lines produced large quantities of seeds under drought and natural conditions. However, the source of the differences must be identified to assess the genetic diversity of genotypes. Therefore, combined analysis was used to dissect the main effects and evaluate links within and between the variation sources. This analysis revealed significant differences in seed yield based on genotypes, environments, and the interactions between them (GEI) (Table 5). The results indicated that environmental conditions were the primary driver of performance variation among sesame genotypes (Table 1) and the difference in the genetic makeup of each line^3,5,43,61,68,70^. However, when genotype-environment interactions are present, the possibility of selection and improvement increases. However, determining its genetic stability becomes more challenging, so we used several methodologies to select the most productive and stable genetic lines.

The AMMI is a valuable statistical model for analyzing GEI and assessing the stability of genotypes^68,71^, to dissect its utility. As a result, we used AMMI (Table 6) and GGE (Fig. 3) models to evaluate the interaction and select the most stable lines. The AMMI model explained (PCA1 and PCA2) 64.71% of the variation for Y, while the GGE biplot explained 76.7% of the variation for Y, indicating that the GGE biplot provided more information on the variability than the AMMI model, similar results recorded by Azon et al.^3^. The results of the AMMI model showed that genotypes, environments, and their interaction have a significant and approximately equal effect on seed yield. However, Azon et al.^3^ found that environments played a central role in regulating seed production. But the AMMI model does not include a quantitative stability measure, which is required to measure and classify genotypes based on yield stability^72^. For this, we used the parametric and non-parameter statistics to estimate genetic stability according to the rank of genotypes^73^. It was observed that the genotype rank changed with changing environments because of crossover interaction^74^. Fortunately, the lines selected based on average seed productivity, the findings of the AMMI and GGE models, and the parametric and non-parametric statistics were all identical, increasing the accuracy of the results. Under drought stress, we found that the selection index containing S⁽^3^⁾, S⁽⁶⁾, NP⁽^2^⁾, NP⁽^3^⁾, and NP⁽^4^⁾ would effectively select the best and stability lines^66,75^. Because there was a positive significant correlation between them (Table 9). Simultaneously, these parameters were significantly positively correlated with other parameters, although they did not have a significant direct positive correlation with yield, which contributes to increasing the selection accuracy. According to this study, four lines (C6-11, C3-8, C9-3, and C6-9) have the ideal balance between genetic stability and high productivity and the lowest role in interaction^76^: ^5^). Therefore, they can be used in many agro-ecological regions of the country, especially those that suffer or may suffer from drought. References^77,78^ recorded similar findings on other crops.

Based on evaluating 28 new sesame lines under various conditions and in water limited (drought) and non-water limited (normal) situations over 18 environments over 4 years, we identified nine lines that yielded higher than the control (i.e., Beagle): C1-5, C2-6, C3-8, C5-8, C6-3, C6-9, C6-11, C9-3 and C9-20. These lines were important for producing seed yield, but the plants had drought tolerance improvements from the control, such as longer root zones that increased water uptake, which allowed for better drought performance than the control. The importance of maintaining genetic diversity for developing crops to improve drought resistance cannot be overstated. The lines discussed in this article could have different combinations of genes that help plants respond and evolve quickly to maintain high productivity. Future improvement is best done with these new lines as parental sources for breeding programs that will add genes that lead to high productivity under drought stress. Additionally, different lines were identified as valuable genetic resources for particular soil conditions including, clay soils (C2-2, C9-6, C6-11, C9-3, C5-8, and C2-6) and sandy soils (C6-3, C9-7, C6-11, C9-3, C6-9 C5-8, C9-20, and C2-6), which would be useful for purposefully, site-directed hybridization programs. Furthermore, lines C6-11, C5-8 and C2-6 were the most productive in both drought and normal conditions in various environments (highest yield), these lines provided the best potential for hybridization programs. Statistical modeling using AMMI (64.71% variation explained) and GGE (76.7% variation explained) were both important in figuring out genotype x environment interactions and provided evidence that they should address environmental conditions together with genotype, and their interaction for seed yield. Collectively, with parametric and non-parametric stability statistics, were important for identifying stable and high yielding genotypes, lines C6-11, C3-8, C9-3, and C6-9, the best combinations of genetic stability and productivity whose interaction effects can be ignored and cultivated in numerous agro-ecological areas of Egypt, especially those prone to drought.

SCoT-PCR has been effectively used in many investigations to study the genetic relationships between different genotypes^32,34,35,79–82^. As in our results, SCoT-21 gave 88.24%, 0.3580, and 10.03 for P %, PIC and EMR% respectively. Reference^83^ used start codon targeted tags (SCoT) markers to study the genetic diversity and relationships among 53 E. sibiricus strains from China. Also, 25 SCoT primers were utilized to get the relationships and genetic diversity between sex local cultivars of sesame in Saudi Arabia, the recorded P %, PIC, MI, and EMR% were the highest with primer SCoT-21^49^. Their variations represent robust and dependable markers identified within this short, shared segment of plant genes adjacent to the ATG start of translation. Serag et al.^84^ used ten SCoT primers to characterize the genotype of five cultivars and ten heterosis under drought stress, obtained 126 amplified fragments with a 52% polymorphism of 15 Sesamum indicum L. with genetic similarity ranging from 75 to 92%. And the 15 genotypes of sesame examined were divided into two main clusters and two main sub-clusters using a dendrogram. This phylogenetic tree offers a fascinating glimpse into the genetic relationships of these sesame lines. The stark separation of Shandweel underscores its unique genetic identity, potentially representing a distinct lineage or an older variety preserved through traditional agricultural practices. Its position raises questions about its origins and the evolutionary forces that have led to its divergence from the other lines. Could it possess unique traits that have been lost in more modern varieties? Further investigation into Shandweel’s characteristics could uncover valuable genetic resources for breeding programs.

DNA repair helicase XPD gene, through its role in the nucleotide excision repair (NER) pathway, has hand in drought stress tolerance by helping plants maintain genome in stress-induced DNA damage facing^85^. Environmental stress, including drought, can induce DNA damage, and efficient repair mechanisms are pivotal for plant survival^86^. Helicases have emerged as important players in plant stress tolerance^87^.

The malonyl-coenzyme: anthocyanin 5-O-glucoside-6′′′-O-malonyl transferase-like gene is essential for producing diverse and stable anthocyanin pigments, which contribute to the visual appeal and physiological functions of plants.

The clustering of Sohag with lines C1-5, C1-6, C1-8, and C1-9 suggests a shared ancestry or breeding history. This group may represent a particular lineage with desirable traits that have been selected for over time. Analyzing the specific traits of these lines could reveal the underlying reasons for their genetic similarity.

The intricate relationships within Group 2 highlight the complex interplay of genetic factors that shape sesame diversity. The presence of subclusters within this group suggests that even within a broader genetic lineage, there is considerable variation. This diversity is a valuable resource for breeders, offering a range of genetic material to select from when developing new varieties with improved characteristics.

Furthermore, this tree emphasizes the importance of conserving sesame germplasm. Each line represents a unique combination of genetic information, shaped by evolutionary processes and human selection. Preserving this diversity is crucial for ensuring the long-term resilience of sesame cultivation in the face of challenges such as climate change and emerging diseases.

While this SCoT marker-based analysis provides valuable insights, combining it with other molecular markers and phenotypic data would offer a more comprehensive understanding of sesame diversity. Analyzing traits like growth habits, seed size, and disease resistance alongside genetic data would allow for a more nuanced interpretation of the evolutionary relationships and adaptive strategies of these sesame lines.

References^49,84^ reported similar results. As a result, a hybridization program for drought-affected or drought-prone lands can be carried out by selecting genetically distinct parents with stable genetics and high productivity. Four lines (C2-2, C5-8, C6-11, and C9-3) were selected as genetically divergent parents that are distinguished by their higher yields and more stability. Finally, this research suggested three lines (C6-11, C3-8, and C6-9) for initial crop experiments in Northern and Western Egypt, particularly in areas with limited water resources. Lines (C2-2, C9-6, C6-11, and C3-8) were recommended to cultivate in the north-central, three lines (C5-8, C2-6, and C3-8) in the northern, and seven lines in the western delta in Egypt. Similar findings recorded by^64,88,89^.

The current study provides useful resources for sesame breeding programs, particularly in developing drought-resistant varieties. The identification of elite lines like C2-2, C5-8, C6-11, and C9-3, which exhibit both high yield and genetic stability under drought stress, offers improved resources for breeders.

These high-yielding lines can serve as parental lines in crosses with other sesame varieties, facilitating the introgression of drought tolerance genes.

## Conclusion

The current study investigated the possibility of using different methods to estimate the genetic stability of sesame genotypes under drought conditions to identify the best genetic sources and using SCoT-PCR markers to study phylogenetic relationships. The results of this study confirmed that AMMI and GGE biplot were sufficient to detect interactions between genotypes and environments. It was found that the lines selected based on AMMI and GGE biplot and parameters and non-parametric statistics matched the lines that were distinguished in yield, indicating that they combined genetic stability and high productivity. This study proposed a selection index containing yield and non-parametric statistics that would effectively select the best genetic resources under drought stress. And the SCoT molecular marker proved its efficiency in determining the genetic similarities and clustering patterns of 30 sesame genotypes. The present study identified suitable sesame lines for drought-affected areas, in addition to identifying lines for evaluation trials at several sites. The overall results showed that the lines C2-2, C5-8, C6-11, and C9-3 had desirable values for seed production as well as the ability to stabilize genetic phenotypes under water stress conditions and can be used as promising genetic resources for developing drought-resistant sesame varieties.

To further enhance sesame drought tolerance, future research should prefer identifying the specific responsible genes through mapping and physiological studies to understand the adaptation mechanisms. Multi-location trials are essential to validate the stability of selected lines across diverse environments, while investigating marker-assisted and genomic selection will accelerate the breeding program.

These advancements will significantly contribute to food security in drought-prone regions by increasing the availability of nutritious food and oil, improving farmer livelihoods, and stabilizing crop production to mitigate crop failure.

## Data Availability

The data that support the findings of this study are available from the corresponding author upon reasonable request.
